# Survey and analysis of kindergarten teachers’ knowledge, attitude, and practice (KAP) of unintentional injury prevention and first aid for children: A cross-sectional study in Mianyang, China

**DOI:** 10.1097/MD.0000000000046776

**Published:** 2026-01-09

**Authors:** Zhi Zeng, Yuqi Shen, Qingping Ma, Xuemei Deng, Mei He, Li Wan

**Affiliations:** a Intensive Care Unit, Mianyang Central Hospital, Affiliated with the School of Medicine, University of Electronic Science and Technology, Mianyang, China; b School of Nursing, North Sichuan Medical College, Nanchong, China; c Beichuan Qiang Autonomous County Institute of Educational Development, Mianyang, China; d Mianyang City Children’s Palace Kindergarten, Mianyang, China.

**Keywords:** children, kindergarten teachers, prevention and first aid, unintentional injury

## Abstract

This study aims to investigate the current status of knowledge, attitudes, and practices (KAP) among kindergarten teachers in Mianyang regarding the prevention and first aid of unintentional injuries for children and analyze the associated influencing factors, to provide a reference for enhancing teachers’injury prevention capabilities and developing targeted training strategies. Based on the KAP theory and drawing upon previous literature and research findings, this study developed a questionnaire to investigate the level of KAP among kindergarten teachers regarding the prevention and first aid of unintentional injuries for children. The questionnaire consisted of 32 items across 3 dimensions: knowledge (11 items), attitude (8 items), and practice (13 items). A multistage stratified random sampling method was employed to select kindergarten teachers in Mianyang City. The questionnaire was distributed online to the targeted teachers via the Wenjuanxing platform. Univariate analysis and multiple linear regression analysis were used to identify the influencing factors. A total of 277 valid questionnaires were received for an effective recovery rate of 92.30%. Among the kindergarten teachers who completed the survey, the total KAP score was 96.71 ± 8.72, with a scoring rate of 83.4%. The scores for each dimension from highest to lowest were: attitude dimension (38.28 ± 2.25) with a scoring rate of 95.7%; practice dimension (50.42 ± 7.12) with a scoring rate of 77.6%; and knowledge dimension (7.99 ± 1.82) with a scoring rate of 72.6%. Multiple linear regression analysis showed that marital status (*P* = .044, 95% CI: −10.714 to −0.145), childbearing history (*P* = .008, 95% CI: 2.016 –13.372), working years (*P* = .005, 95% CI: 0.391 –2.208), participation in training related to unintentional injury prevention and first aid for children (*P* < .001, 95% CI: 3.547 –7.462), and experience with unintentional injury incidents involving children (*P* = .030, 95% CI: 0.235 –4.548) were influencing factors for kindergarten teachers’ KAP level in preventing unintentional injuries and providing first aid for children (*P* < .05). The KAP levels among kindergarten teachers in Mianyang regarding unintentional injury prevention and first aid for children need improvement. Among these, the knowledge dimension scored relatively low, indicating a need to strengthen training and learning in unintentional injury prevention and first aid for children, to promote the healthy development of children.

## 1. Introduction

Unintentional injury refers to physical harm resulting from external, sudden, unintended, and non-disease-related events.^[1]^ According to the World Report on Child Injury Prevention, unintentional injuries are the greatest threat to children’s health and life, one of the leading causes of disability, and have become a serious global public health issue.^[2]^ In China, unintentional injury is the leading cause of death among children, with over 50,000 children dying from such injuries annually, accounting for 40% to 50% of all child deaths.^[3]^ Among these, preschool children – due to their rapid physical development, active nature, limited cognitive abilities, and lack of self-protection awareness and skills – are at high risk for unintentional injuries.^[4]^ Kindergartens are common settings for such injuries, and kindergarten teachers are the closest adults to children in these environments. On 1 hand, teachers are directly involved in preventing and providing first aid for unintentional injuries in kindergartens; on the other hand, they can disseminate knowledge about unintentional injury prevention to children and parents through home-kindergarten partnerships. Their role in preventing injuries, reducing their incidence, severity, disability, and mortality, and promoting children’s safety and healthy development cannot be overlooked.^[5]^ Some studies have pointed out that issues such as subjective judgment and negligence among preschool teachers may exacerbate the occurrence of unintentional injuries.^[6]^ Currently, the majority of domestic and international research on unintentional injuries has concentrated on creating safe home environments and enhancing parental safety awareness and knowledge,^[7,8]^ with limited exploration from the perspective of kindergarten teachers.

The knowledge, attitude, and practice (KAP) theory posits that the accumulation of knowledge serves as the foundation for attitude change, which in turn facilitates behavioral transformation.^[9]^ This theory is frequently applied in questionnaire design to assess the current status of KAP among specific populations in a given region, and to develop intervention measures based on the results to promote the formation of positive behaviors.^[10]^ Mianyang, as one of the areas severely affected by the “5·12” catastrophic earthquake, had its residents generally experience this disaster. This unique experience prompted them to proactively learn first aid knowledge and skills through various channels, thereby enhancing their first aid awareness and willingness. However, while previous studies have focused on the family rescue willingness of Mianyang residents^[11]^ and children’s awareness of disaster prevention and mitigation as well as their self-rescue and mutual aid capabilities,^[12]^ there has been no systematic investigation into the KAP status of kindergarten teachers in the region regarding unintentional injury prevention and first aid for children. Therefore, this study, framed by the KAP theory, conducts a survey among kindergarten teachers in Mianyang City. It aims to analyze their KAP levels and current practices concerning unintentional injury prevention and first aid for children, and to explore the related influencing factors. Based on these findings, targeted intervention strategies will be developed to provide scientific evidence and practical guidance for kindergartens to carry out systematic and comprehensive work on unintentional injury prevention and first aid.

## 2. Materials and methods

### 2.1. Study design

This study was designed and conducted as a cross-sectional study, and the reporting of this study was done following the Strengthening the Reporting of Observational Studies in Epidemiology statement. We hypothesize that these findings may help scholars identify the KAP levels among kindergarten teachers in Mianyang, China, regarding unintentional injury prevention and first aid for children. The results may also provide a reliable basis for developing targeted training strategies in Mianyang, China. The specific objectives include: to assess the knowledge level of kindergarten teachers concerning unintentional injury prevention and first aid for children; to evaluate the attitude level of kindergarten teachers toward unintentional injury prevention and first aid for children; to investigate the practice level of kindergarten teachers in unintentional injury prevention and first aid for children; and to identify the multiple factors influencing the KAP levels of kindergarten teachers regarding unintentional injury prevention and first aid for children.

### 2.2. Participants

In November 2024, a multistage stratified random sampling method was used to select kindergarten teachers in Mianyang City as the study participants. Based on per capita gross domestic product, the districts/counties of Mianyang were divided into 3 levels: high, medium, and low. One district/county was randomly selected from each level, namely Fucheng District, Beichuan County, and Youxian District. From each selected district/county, 1 public and 1 private kindergarten were chosen from both urban and rural areas, totaling 12 kindergartens. All kindergarten teachers in these selected kindergartens were included as survey participants. Written informed consent was obtained from all participating kindergarten teachers. According to the principle that the sample size be at least 5 to 10 times the number of independent variables,^[13]^ given the 31 variables in this study and an assumed 10% rate of invalid samples, the resulting sample size should be 192 to 384 cases.

### 2.3. Data collection tools and methods

#### 2.3.1. Data collection tool

This study utilized a self-developed, culturally tailored assessment tool to evaluate the knowledge, attitude, and practice (KAP) levels of kindergarten teachers in Mianyang City, China, regarding unintentional injury prevention and first aid for children. The questionnaire consisted of 2 parts – general information questionnaire: this section included 12 items covering the kindergarten teachers’ gender, age, marital status, childbearing history, educational background, major, kindergarten location, type of kindergarten, years of work experience, average monthly income, prior experience with unintentional injury incidents involving children, and participation in training related to unintentional injury prevention and first aid for children. KAP questionnaire: guided by the KAP theoretical framework, this questionnaire was developed based on a review of literature and existing research tools related to unintentional injury prevention and first aid for children,^[14,15]^ and further adapted in accordance with the current situation of unintentional injury prevention and first aid for children in China.^[16]^ The questionnaire encompassed 3 dimensions – knowledge, attitude, and practice – with a total of 32 items. Knowledge dimension (11 items): this section assessed knowledge about unintentional injury prevention and first aid for children, including topics such as burns and scalds, falls and sprains, and foreign body airway obstruction. Each correct response was scored as 1 point, and each incorrect response as 0 points. The total score for this dimension ranged from 0 to 11, with higher scores indicating better knowledge of unintentional injury prevention and first aid for children. Attitude dimension (8 items): this section used a 5-point Likert scale, with responses ranging from “Strongly disagree” to “Strongly agree,” scored from 1 to 5 points respectively. The total score for this dimension ranged from 8 to 40, with higher scores reflecting more positive attitudes toward unintentional injury prevention and first aid for children. Practice dimension (13 items): this section also used a 5-point Likert scale, with responses ranging from “Never” to “Always,” scored from 1 to 5 points. The total score for this dimension ranged from 13 to 65, with higher scores indicating better practice behaviors related to unintentional injury prevention and first aid for children. Prior to formal distribution, a pilot survey was conducted with 40 kindergarten teachers. The reliability of the questionnaire was tested using SPSS 27.0 software. Results showed that the average completion time ranged from 120 to 300 seconds, and the Cronbach’s α coefficient was 0.854, indicating good internal consistency reliability.

The total questionnaire score was calculated as the sum of the scores from the knowledge, attitude, and practice dimensions, yielding a total score range of 21 to 116 points. A higher score indicates a higher level of KAP among kindergarten teachers regarding unintentional injury prevention and first aid for children. Drawing on relevant studies,^[17]^ the total score and the scores for each dimension (knowledge, attitude, and practice) were converted into percentage scores using the following formula: Score rate (%) = (Average dimension score/Total possible dimension score) × 100%. Based on the score rates, the levels were classified as follows: high level (>85%), moderate level (60–85%), and low level (<60%).

#### 2.3.2. Data collection method

Questionnaire data were collected by distributing a quick response code link via the WeChat platform. After obtaining approval from each participating institution, the researchers sent the questionnaire link to a WeChat group composed of heads of various kindergartens in Mianyang City. The purpose, significance, and instructions for completing the questionnaire were explained to the heads, who then forwarded the link to the kindergarten teachers in their respective institutions. Throughout the survey process, members of the research team promptly addressed any questions via WeChat or telephone to ensure the quality of responses. The questionnaire did not require the inclusion of personal identifying information such as names. It was configured to allow only 1 submission per internet protocol address, and all items were set as mandatory. Participants began filling out the questionnaire only after selecting the option to agree to participate in the survey.

### 2.4. Data analysis

Two researchers independently organized and entered the questionnaire data. After cross-checking and conducting a consistency check, an Excel database was established. Data were statistically analyzed using IBM SPSS 27.0 (IBM Corp., Armonk). The statistical methods included descriptive statistical analysis, 1-way analysis of variance, and multiple linear regression analysis. A *P*-value of <.05 was considered statistically significant.

### 2.5. Ethics and research approval

This research protocol was approved by the Ethics Committee of Mianyang Central Hospital (Approval No. S20240227-01). All participants were informed of the study’s purpose, and confidentiality of their personal information was ensured. They were also informed of their right to withdraw from the study at any time without consequences.

## 3. Results

### 3.1. Basic characteristics

**Figure 1.**
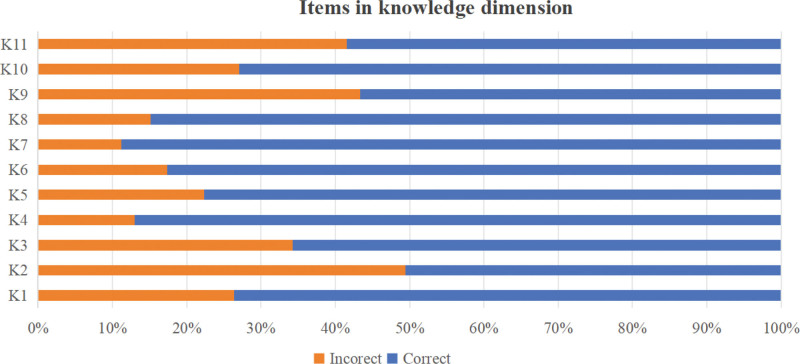
Scores of each item in the knowledge dimension regarding unintentional injury prevention and first aid for children among kindergarten teachers.

**Figure 2.**
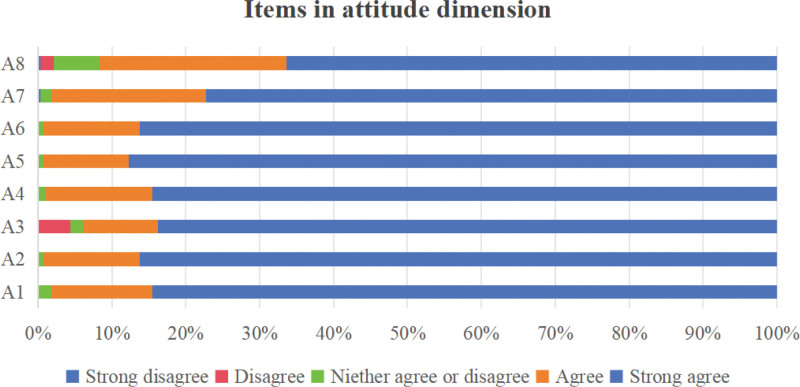
Scores of each item in the attitude dimension regarding unintentional injury prevention and first aid for children among kindergarten teachers.

**Figure 3.**
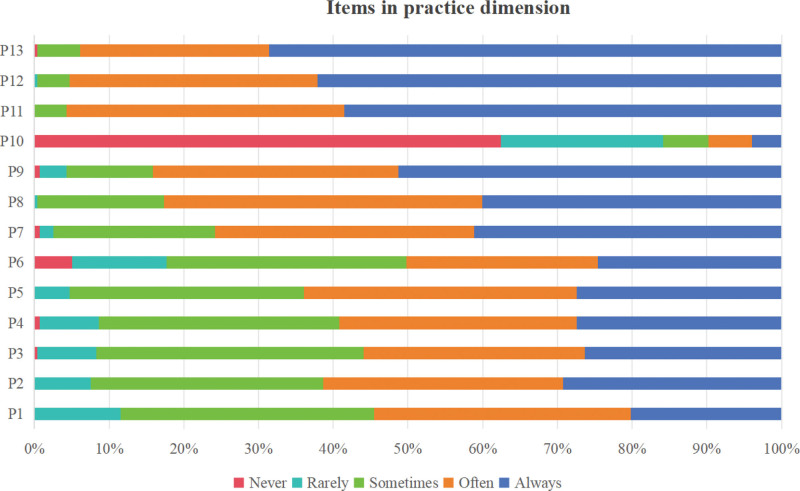
Scores of each item in the practice dimension regarding unintentional injury prevention and first aid for children among kindergarten teachers.

A total of 300 questionnaires were distributed in this study, and 286 were collected. After excluding questionnaires completed in <120 seconds and those in which all items were answered with the same option, 277 valid questionnaires were obtained, yielding an effective response rate of 92.3%. The total KAP score of the 277 kindergarten teachers was (96.03 ± 8.65), with a scoring rate of 83.4%, indicating a moderate level. The dimension scoring rates, ranked from highest to lowest, were attitude, practice, and knowledge, as detailed in Table 1. Additionally, Figure 1 presents the average scores for each item in the knowledge dimension of unintentional injury prevention and first aid for children among the 277 kindergarten teachers. Similarly, Figure 2 displays this information for the attitude dimension, while Figure 3 illustrates the corresponding data for the practice dimension. (For specific details regarding each item, please refer to Supplementary Material 1, Supplemental digital Content, https://links.lww.com/MD/R82.)

**Table 1:** KAP scores of kindergarten teachers regarding unintentional injury prevention and first aid for children (n, %).

Dimension	Score (*x̄* ± *s*)	Scoring rate (%)
Knowledge score	7.99 ± 1.82	72.6
Attitude score	38.28 ± 2.25	95.7
Practice score	50.42 ± 7.12	77.6
Total KAP score	96.71 ± 8.72	83.4

KAP = knowledge, attitude, and practice.

### 3.2. Comparison of KAP scores regarding unintentional injury prevention and first aid for children among kindergarten teachers with different characteristics

Using the general demographic information of kindergarten teachers as independent variables and their KAP scores regarding unintentional injury prevention and first aid for children as dependent variables, univariate analysis revealed statistically significant differences in KAP scores based on marital status, history of childbirth, years of work experience, monthly income, prior experience with child unintentional injury events, and participation in training programs related to unintentional injury prevention and first aid for children (*P* < .05). No statistically significant differences were observed for the remaining indicators (*P* > .05; for details, see Table 2).

**Table 2:** Comparison of KAP scores on unintentional injury prevention and first aid for children among kindergarten teachers with different characteristics (*x̄* ± *s*).

Variable	Number of participants	KAP score (*x̄* ± *s*)	*t*/*F* value	*P*-value
Gender			−0.196	.845
Male	6 (2.2%)	96.00 ± 10.00		
Female	271 (97.8%)	96.70 ± 8.70		
Age (yr)			0.439	.645
<30	129 (46.6%)	96.19 ± 8.76		
30–40	116 (41.9%)	97.03 ± 8.76		
>40	32 (11.6%)	97.50 ± 8.41		
Marital status			3.609	.014
Unmarried	60 (21.7%)	94.00 ± 8.80		
Married	214 (77.3%)	97.52 ± 8.54		
Divorced	2 (0.7%)	96.00 ± 1.41		
Widowed	1 (0.4%)	82.00		
Childbearing history			−3.026	.003
No children	61 (22.0%)	93.75 ± 8.58		
Has children	216 (78.0%)	97.52 ± 8.58		
Education level			3.152	.077
College and below	35 (12.6%)	94.26 ± 6.64		
Bachelor and above	242 (87.4%)	97.04 ± 8.92		
Major			2.369	.096
Preschool education	190 (68.6%)	97.32 ± 9.01		
Other education majors	82 (29.6%)	95.61 ± 7.90		
Medical-related majors	5 (1.8.0%)	90.60 ± 6.23		
Kindergarten location			1.949	.052
Urban	143 (51.6%)	97.67 ± 8.26		
Rural	134 (48.4%)	95.64 ± 9.07		
Kindergarten type			0.042	.966
Public	103 (37.2%)	96.72 ± 8.53		
Private	174 (62.8%)	96.67 ± 8.83		
Work experience (yr)			5.015	.002
<3	43 (15.5%)	93.21 ± 7.73		
3–5	46 (16.6%)	94.52 ± 9.46		
6–10	80 (28.9%)	98.44 ± 8.13		
>10	108 (39%)	97.7 ± 8.68		
Monthly income (Renminbi)			3.417	.018
<3000	119 (43.0%)	95.6 ± 8.22		
3000–5000	98 (35.4%)	98.62 ± 8.86		
5001–10,000	33 (11.9%)	94.09 ± 9.88		
>10,000	27 (9.8%)	97.67 ± 7.45		
Participation in training on unintentional injury prevention and first aid for children			−5.363	<.001
No training	174 (62.8%)	94.63 ± 8.49		
Received training	103 (37.2%)	100.17 ± 7.97		
Experience with child unintentional injury events			−2.488	.013
No experience	73 (26.4%)	94.53 ± 8.44		
Has experience	204 (73.6%)	97.46 ± 8.69		

KAP = knowledge, attitude, and practice.

### 3.3. Linear regression analysis of factors influencing kindergarten teachers’ KAP regarding unintentional injury prevention and first aid for children

The total score of the KAP questionnaire regarding unintentional injury prevention and first aid for children among kindergarten teachers was used as the dependent variable. Variables with *P* < .05 from the comparisons in Table 2 were included as independent variables. The results indicated that marital status, history of childbirth, years of work experience, participation in training programs on unintentional injury prevention and first aid for children, and prior experience with child unintentional injury events were all significant influencing factors on the KAP level of kindergarten teachers (*P* < .05; see Table 3 for details).

**Table 3:** Multiple linear regression analysis of kindergarten teachers’ KAP regarding unintentional injury prevention and first aid for children (n = 277).

Variable	*B*	SE	β	*t*	*P*	95% CI	
						Lower limit	Upper limit
(Constant)	92.158	3.292		27.992	<.01	85.676	98.64
Marital status	−5.429	2.684	−0.278	−2.023	.044	−10.714	−0.145
Childbearing history	7.694	2.884	0.367	2.668	.008	2.016	13.372
Work experience	1.299	0.461	0.162	2.817	.005	0.391	2.208
Monthly income	0.371	0.501	0.041	0.741	.459	-0.615	1.357
Participation in training on unintentional injury prevention and first aid for children	5.505	0.994	0.306	5.537	<.01	3.547	7.462
Experience with child unintentional injury events	2.391	1.095	0.121	2.183	.030	0.235	4.548

*R*^*2*^ = 0.177, after the adjustment *R*^*2*^ = 0.159; *F* = 9.709, *P <* .001.

CI = confidence interval, KAP = knowledge, attitude, and practice, SE = standard error.

## 4. Discussion

### 4.1. Kindergarten teachers’ KAP regarding unintentional injury prevention and first aid for children needs improvement

This study shows that the total KAP score of kindergarten teachers in Mianyang City regarding unintentional injury prevention and first aid for children was (96.71 ± 8.72), with a scoring rate of 83.7%. The scores of each dimension, ranked from highest to lowest, were as follows: attitude dimension (38.28 ± 2.25), scoring rate 95.7%; practice dimension (50.42 ± 7.12), scoring rate 77.6%; and knowledge dimension (7.99 ± 1.82), scoring rate 72.6%. Overall, the KAP level of kindergarten teachers in Mianyang City regarding unintentional injury prevention and first aid for children was moderate. This finding is consistent with the results reported by Narantsetseg et al,^[18]^ further reinforcing the view that kindergarten teachers have significant deficiencies in unintentional injury prevention and first aid for children. Notably, the knowledge dimension scored relatively low, suggesting that targeted training and learning in this area should be strengthened.

In terms of the knowledge dimension, this study found that kindergarten teachers demonstrated a good grasp of preventing common unintentional injuries such as electric shock, falls, sprains, and burns. However, their performance was inadequate in first aid knowledge areas, including the Heimlich maneuver, cardiopulmonary resuscitation (CPR), and drowning first aid. This finding is consistent with the survey results of Maalim et al^[19]^ on kindergarten teachers’ KAP regarding choking first aid. This imbalance in knowledge acquisition can be attributed to 2 key factors. On the 1 hand, the rapid development of modern technology and the widespread availability of online media have enabled basic prevention knowledge to be disseminated quickly through multiple channels, making it relatively easy for kindergarten teachers to access and master these simpler concepts.^[20,21]^ On the other hand, content such as the Heimlich maneuver, CPR, and drowning first aid is highly specialized and operationally complex. Mastery of these skills requires not only theoretical learning but also systematic guidance from professionals and repeated practical training.^[22,23]^ Based on these findings, it is recommended that educational authorities formally incorporate training on unintentional injury first aid for children into the teacher training system.^[24]^ Diversified training approaches should be adopted, such as developing gamified teaching modules and interactive assessment mechanisms offline,^[25]^ and utilizing virtual reality technology for immersive remote training online.^[26]^ These methods can comprehensively enhance kindergarten teachers’ professional competence and practical skills in providing first aid for unintentional injuries in children. This will contribute to strengthening child safety measures.^[27]^

In terms of the attitude dimension, although most kindergarten teachers held positive attitudes toward unintentional injury prevention and first aid for children, the item “willingness to provide emergency rescue during unintentional injury incidents involving children” received a relatively low score. This finding aligns with the results of Chen et al^[28]^ on emergency rescue behaviors among laypersons. Comparative data indicate that the out-of-hospital cardiopulmonary resuscitation (CPR) rate in mainland China is only 4.5%, significantly lower than rates in the United States (46.1%), Canada (29%), Sweden (46–73%), Japan (32.2%), and Australia (21.2%).^[29]^ Clearly, there is an urgent need for broader first aid education and training in China. However, the question of whether kindergarten teachers are willing to provide emergency rescue during unintentional injury incidents involving children is more complex. Their willingness depends not only on mastery of first aid techniques but, more critically, on their rescue beliefs, psychological preparedness, and self-efficacy. Key barriers to proactive rescue include lack of first aid knowledge,^[30]^ fear of causing secondary harm through improper operation, and psychological concerns such as infection risk (e.g., during mouth-to-mouth resuscitation).^[31]^ To effectively enhance kindergarten teachers’ willingness and capacity for first aid, modern communication technologies should be leveraged to establish video-assisted first aid systems, enabling teachers to receive real-time guidance from professional dispatchers during rescue procedures and thereby reduce fear of operational errors.^[32]^ Additionally, targeted training content based on first aid guidelines should be developed to improve emergency response capabilities and self-efficacy for common childhood unintentional injuries through simulation drills.^[33]^ Furthermore, public education campaigns should promote the concept that “imperfect first aid is better than no first aid” and disseminate legal knowledge protecting bystanders from civil or criminal liability when performing CPR,^[34]^ helping kindergarten teachers overcome psychological barriers and encouraging decisive action in emergencies.^[35]^ Only through coordinated efforts in technical support, education and training, and psychological empowerment can the first aid attitudes of kindergarten teachers be fundamentally transformed, enhancing their emergency response capabilities in unintentional injury incidents involving children.

In terms of the practice dimension, kindergarten teachers scored lower on items such as “regularly updating safety education teaching content” and “innovating safety education teaching methods.” This reflects a lack of investment and innovation in safety education among kindergarten teachers, which is consistent with the findings of Hao et al.^[36]^ To address this issue, it is recommended that kindergarten teachers dynamically adjust and update teaching content in accordance with the cognitive developmental levels of children across different age groups and the epidemiological characteristics of unintentional injuries in various regions, thereby enhancing its practical relevance and acceptability. At the same time, diverse teaching methods – such as scenario simulation, gamified instruction, multimedia teaching, and interactive approaches – should be actively adopted to effectively improve children’s engagement, learning interest, and active participation.^[37]^This survey also revealed that kindergarten teachers scored relatively low on the item regarding “provision of unintentional injury first aid supplies in kindergartens,” indicating a significant gap between China’s kindergartens and those in developed countries in terms of first aid resource allocation. For instance, in Canada, the proportion of schools equipped with first aid supplies across all registered sites has reached 72.5%.^[38]^ In contrast, the availability rate in Chinese kindergartens remains below this level. Moving forward, there is an urgent need to strengthen the standardized configuration of first aid supplies in kindergartens, increase their coverage rate,^[39]^ and install prominent signage at storage locations to ensure that teachers can quickly locate and access them during emergencies, thereby effectively enhancing emergency response capabilities.^[40]^

### 4.2. Kindergarten teachers’ KAP regarding unintentional injury prevention and first aid for children are influenced by multiple factors

Multiple linear regression analysis revealed that kindergarten teachers who were married, had a history of childbirth, possessed longer years of work experience, had participated in unintentional injury prevention training, or had experienced unintentional injury events demonstrated higher levels of KAP regarding unintentional injury prevention and first aid for children. The possible reasons are as follows: married kindergarten teachers and those with a history of childbirth often assume multiple roles as both educators and caregivers, leading to heightened awareness of unintentional injuries among young children. This finding aligns with the research by Machin et al.^[41]^ Therefore, kindergartens should fully leverage the strengths of this group by encouraging them to take a leading role in campus safety culture development. Additionally, through experience sharing and peer support, they can help enhance the safety awareness of other teachers, fostering a collective commitment to child safety.^[42]^ Teachers with longer work experience have accumulated rich professional expertise and life experience, enabling them to better understand the importance of unintentional injury prevention and first aid, and to implement more comprehensive preventive measures. Similar findings were reported by Filemban et al.^[43]^ Based on this, kindergartens should establish mentorship or experience sharing mechanisms, encouraging senior teachers to guide new staff and transform valuable experience into replicable and scalable safety education models. Regular experience exchange meetings should also be organized to promote knowledge sharing and capacity building.^[44]^ Teachers who have experienced unintentional injury events or participated in relevant training tend to actively focus on key knowledge points, thereby enhancing their emergency response capabilities. This further corroborates the KAP theory’s emphasis on the critical role of knowledge accumulation in shaping attitudes and guiding behaviors.^[45]^ Moving forward, kindergartens should integrate unintentional injury prevention and first aid training into teachers’ continuing education systems. Regular specialized training and emergency drills should be organized, covering the latest prevention guidelines, first aid protocols, and legal protections to ensure continuous knowledge updates and skill reinforcement.^[46]^

### 4.3. Limitations

First, although a stratified sampling method was employed, the sample was drawn exclusively from Mianyang City, China. Given China’s vast geographical expanse, significant disparities exist across regions in terms of educational resource allocation, teacher training systems, implementation of child injury prevention policies, and teaching practices. Consequently, a sample from a single city cannot fully represent the overall status of kindergarten teachers’ knowledge, attitude, and practice (KAP) regarding unintentional injury prevention and first aid for children nationwide. This geographical limitation constrains the generalizability and representativeness of the findings. Future studies should expand the sample coverage to include educational institutions from regions with varying levels of economic development across the eastern, central, and western parts of China, or adopt a multicenter research design to enhance the external validity and generalizability of the conclusions. Second, in terms of data collection, the questionnaire was self-administered, rendering the self-reported content susceptible to recall bias and social desirability bias. Although the study implemented anonymity and confidentiality measures to mitigate these biases, their influence cannot be entirely eliminated. Future research could consider adopting a mixed-methods approach, integrating quantitative and qualitative analyses, supplemented by objective measures (such as actual first aid skill assessments) or behavioral observations, to improve the accuracy and credibility of the data, thereby enabling a more comprehensive evaluation of the current KAP status among kindergarten teachers concerning unintentional injury prevention and first aid for children.

## 5. Conclusion

Reducing the incidence of unintentional injuries among children and promoting their healthy growth are key priorities in current public health efforts. As supervisors and educators in children’s daily school life, kindergarten teachers bear significant responsibility for safeguarding children’s health and safety. Therefore, it is essential to investigate the KAP level of kindergarten teachers regarding unintentional injury prevention and first aid for children, along with its influencing factors, in order to guide kindergartens in advancing related work in this field. This study reveals that the KAP level of kindergarten teachers in Mianyang City concerning unintentional injury prevention and first aid is suboptimal. Marital status, history of childbirth, years of work experience, participation in unintentional injury-related training, and prior experience with unintentional injury events are all factors influencing teachers’ KAP in this domain. Moving forward, it is necessary to develop and implement efficient training programs tailored to the characteristics and influencing factors of kindergarten teachers, in order to effectively enhance their KAP regarding unintentional injury prevention and first aid, thereby preventing the occurrence of unintentional injuries and genuinely promoting the healthy development of children.

## Acknowledgments

The authors appreciate all the respondents who cooperated with this survey, as well as the managers and representatives of each of the collaborating centers who assisted in the survey.

## Author contributions

**Data curation:** Zhi Zeng, Yuqi Shen, Qingping Ma, Xuemei Deng, Mei He.

**Writing – original draft:** Zhi Zeng, Yuqi Shen.

**Writing – review & editing:** Li Wan.

## Supplementary Material


