# Supplementary material for: Survey and analysis of kindergarten teachers’ knowledge, attitude, and practice (KAP) of unintentional injury prevention and first aid for children: A cross-sectional study in Mianyang, China
Source: Medicine (Baltimore). 2026 Jan 9;105(2):e46776. doi: 10.1097/MD.0000000000046776 (PMC12795092; doi:10.1097/MD.0000000000046776)
Supplement: Supplementary file 1 [file medi-105-e46776-s001.docx]

Supplementary Material 1 Items and Content of the KAP Questionnaire

| Items | content |
| --- | --- |
| K1 | When performing cardiopulmonary resuscitation on a child, the chest compression depth should be at least 1/3 of the anteroposterior diameter of the chest |
| K2 | When a child is bitten by an animal, the wound should be immediately cleaned with water and soap |
| K3 | After a drowning child is rescued from the water, blockages in the mouth and nose should be cleared first to maintain airway patency |
| K4 | Prominent warning signs should be posted around electrical sockets in the classroom |
| K5 | When there is water on the classroom floor, it should be wiped immediately or warning signs should be placed |
| K6 | Simulated food props used for teaching should be locked in cabinets |
| K7 | When a child’s finger is cut by a sharp object and bleeding, pressure can be applied to the wound to stop bleeding |
| K8 | When a child has a sprain, ice application can be used to reduce swelling |
| K9 | When blisters appear after a child is scalded, they should be rinsed with clean water and then covered with loose protective dressings |
| K10 | When performing the Heimlich maneuver, the child should sit on your curved thighs, keeping their body leaning forward, head slightly lowered, and mouth open |
| K11 | When a child suffers from gas poisoning, they should first be taken away from the poisoning site |
| A1 | Willing to implement emergency rescue when unintentional child injury incidents occur |
| A2 | It is necessary to equip the kindergarten area with items related to unintentional child injury first aid |
| A3 | It is necessary to cooperate with relevant social departments (traffic police, fire department, etc.) to jointly carry out safety education work |
| A4 | It is necessary to conduct training activities on unintentional child injury prevention and first aid |
| A5 | It is necessary to invite parents to jointly participate in child safety education work |
| A6 | It is necessary to regularly innovate safety education teaching methods |
| A7 | It is necessary to learn knowledge and skills related to unintentional child injury prevention and first aid |
| A8 | It is necessary to have a kindergarten doctor in the kindergarten |
| P1 | Participating in training related to unintentional child injury prevention and first aid |
| P2 | Reporting children’s unsafe behaviors to parents |
| P3 | Reporting children’s abnormal physical conditions to the kindergarten doctor |
| P4 | Innovating child safety education teaching methods |
| P5 | Not browsing mobile phones while supervising children |
| P6 | Learning content related to unintentional child injury prevention and first aid |
| P7 | Stopping children’s dangerous behaviors and providing education |
| P8 | Equipping the kindergarten area with items related to unintentional child injury first aid |
| P9 | Checking whether children are carrying sharp objects into the kindergarten area |
| P10 | Discussing topics related to unintentional child injury prevention and first aid with others |
| P11 | Regularly updating child safety education teaching content |
| P12 | Participating in training related to unintentional child injury prevention and first aid |
| P13 | Educating parents to pay attention to unintentional child injury prevention and first aid |
